# Inbreeding in a Fission–Fusion Equid Decreases Following an Increase in the Number and Redistribution of Water Sources

**DOI:** 10.1111/mec.70516

**Published:** 2026-08-17

**Authors:** Noa Yaffa Kan‐Lingwood, Alan R. Templeton, Naama Shahar, Daniel I. Rubenstein, Amos Bouskila, Shirli Bar‐David

**Affiliations:** ^1^ Mitrani Department of Desert Ecology Ben‐Gurion University of the Negev, The Swiss Institute for Dryland Environmental & Energy Research, Blaustein Institutes for Desert Research Midreshet Ben‐Gurion Israel; ^2^ Department of Biology Washington University St. Louis Missouri USA; ^3^ Department of Ecology and Evolutionary Biology Princeton University Princeton New Jersey USA; ^4^ Department of Life Sciences Ben‐Gurion University of the Negev Beer‐Sheva Israel

**Keywords:** *Equus hemionus*, fission–fusion, genetic relatedness, inbreeding, inbreeding avoidance, mating patterns, polygyny, resource management, resource‐defence polygyny, water sources

## Abstract

Social structures and habitat configuration influence inbreeding in wild populations. In harem‐forming, female‐defence polygynous equids, social organization promotes inbreeding avoidance, but this has not been studied in fission–fusion, resource‐defence equid species. Because reproduction depends on solitary males establishing territories near resources, shifts in resource distribution are expected to affect mating patterns and inbreeding. We examined inbreeding patterns in the Asiatic wild ass ( *Equus hemionus* ) in the Negev Highlands, Israel (2019–2023), by comparing mating‐pair relatedness (MPR) to all female–male dyads, within each year, and testing whether these patterns changed following a 2020 water source increase and redistribution by increasing the number of new territorial males. We then examined whether females overall tended to mate with veteran (i.e., mating since 2019) or new (i.e., mating since 2020) reproducing males, and compared female–male genetic relatedness between these two groups. Using 2509 faecal samples (2020–2024) genotyped at 625 SNPs, we assigned parentage to 73 offspring and estimated the MPR. Within years, the MPR was higher than random in 2019 and 2020 but declined after the water source manipulation, in 2021–2023. The MPR was higher near the original water source than near the new water sources. Most females (87.3%) mated with new reproducing males, and in 68.3% of cases, new reproducing males were less related to females than were veteran males. Our study suggests that reduced inbreeding is possible in fission–fusion populations when females have extended mating opportunities with territorial males, which can be achieved by expanding the distribution of resources.

## 1 Introduction

Social structures strongly shape mating systems and patterns, with consequences for genetic diversity (Clutton‐Brock 2021). For example, dominance hierarchies, social interactions, sex ratios, and group composition generate reproductive skew (Leimar and Bshary 2022). As a result, higher‐ranked individuals secure disproportionate breeding opportunities relative to lower‐ranked individuals, leading to non‐random partner choice (Feldblum et al. 2021; Holekamp and Strauss 2020) and shaping allele frequencies and inherited traits at both individual and population levels (Parreira and Chikhi 2015; Templeton 2021).

One process linking non‐random mating to genetic diversity is inbreeding; excessive matings between relatives increases homozygosity and the expression of recessive alleles (Berger and Cunningham 1987; Keller and Waller 2002). Several mechanisms can contribute to increased inbreeding. For example: patterns of group structure and social associations that promote interactions within stable family groups, thereby increasing encounters between related males and females (Parreira et al. 2020; Sanderson et al. 2015); or mate choice that tolerates or even prefers mating with kin (e.g., green sea turtles [Dolfo et al. 2025], cichlid fish [Thünken et al. 2007]); or lineage‐biased mating, in which repeated mating with the same partner across years in a polygamous mating system produces inbreeding over generations (Stopher et al. 2012). In addition, decreasing global habitat availability may promote inbreeding by increasing encounters between potential breeders since limited dispersal and spatial kin clustering are strongly affected by habitat configuration (Parreira et al. 2020).

Habitat configuration is a major factor influencing social and mating structures (Auclair et al. 2025; Giuntini and Pedruzzi 2023; He et al. 2019). Among ungulates, for example, females' movement and space use are largely dictated by the distribution of food and water availability, which, in turn, can shape male strategies ranging from female defence to resource territory defence (Bowyer et al. 2020; Corlatti and Lovari 2023; Panebianco et al. 2021). In resource‐defence polygynous systems, both limited defensible resources and restricted areas near the resources may increase the chance of matings among kin, thus leading to increased inbreeding. In contrast, increased resource availability can allow more males to achieve reproductive status, thereby reducing mating with relatives (Giuntini and Pedruzzi 2023; He et al. 2019; Panebianco et al. 2021). However, few empirical studies have linked changing resource conditions to inbreeding patterns, despite growing interest in this topic (Galezo et al. 2022; Giuntini and Pedruzzi 2023; Schultz et al. 2020).

Equids' polygynous social and mating systems provide a good example for studying the relationship between sociality, reproduction, and resource distribution (Emlen and Oring 1977; Rubenstein 1986; Linklater et al. 2000; Szemán et al. 2024). These systems include two major social structures, largely based on the resource defended by the males (Boyd et al. 2016; Klingel 1975, 1977; Rubenstein 1986, 2010). The first is female‐defence polygyny, as in Przewalski and feral horses ( *Equus przewalskii* and *Equus caballus* , respectively) (Boyd et al. 2016; Górecka‐Bruzda et al. 2023), and plains and mountain zebras ( *E. quagga* and *E. zebra* , respectively) (Nuñez et al. 2011). These species form harems consisting of relatively stable social groups composed of one stallion, or occasionally a few (Rubenstein and Nuñez 2009), and several reproductive mares and their offspring (Cameron et al. 2009; Linklater et al. 2000; Rubenstein and Nuñez 2009; Szemán et al. 2024). The stallion defends and monopolizes most of the matings with the females in the group. Lower‐ranked males that do not defend females live in separate bachelor groups, occasionally mating with females from defended groups (Berger 1986; Bowyer et al. 2020; Manning and McLoughlin 2019). Such a system typically occurs in mesic environments, where food and water are more evenly and predictably distributed (Bowyer et al. 2020; Moehlman 2002; Rubenstein 1986).

The second type of equid social system involves resource defence by males and is adopted by Grevy's zebras ( *E. grevyi* ), the African wild ass ( *E. africanus* ), kiang ( *E. kiang* ), the Asiatic wild ass ( *E. hemionus* ), and feral donkeys ( *E. asinus* ) living in arid environments (Boyd et al. 2016; Kaczensky et al. 2025; Klingel 1975; Moehlman 1998; Renan et al. 2018; Rubenstein 1986). In contrast to harem‐forming species, these equids inhabit environments where food and water are patchy and scarce, leading to unstable female associations and fission–fusion grouping dynamics (Klingel 1974; Klingel 1975; Rubenstein 2010; Rubenstein et al. 2015). Because males cannot defend or monopolize unstable female groups, they defend territories near valuable resources that females need, such as food patches and water sources (Bowyer et al. 2020; Boyd et al. 2016; Rubenstein et al. 2015; Saltz et al. 2000).

While inbreeding avoidance is often maintained through various behavioural mechanisms (Pusey and Wolf 1996) and has been documented among harem‐forming equid species through parent–offspring recognition or expulsion at a young age (Berger and Cunningham 1987; Górecka‐Bruzda et al. 2023), the extent to which it is maintained in fission–fusion equid species is less well understood. Although social and mating interactions in these species are highly dependent on resource distribution (Cao et al. 2025; Manning and McLoughlin 2019; Renan et al. 2018; Saltz et al. 2000), it is unknown whether changing patterns of resource availability may modulate inbreeding patterns in fission–fusion equid populations.

Studying social and mating interactions with implications for inbreeding is particularly challenging in elusive fission–fusion species because frequent shifts in group membership complicate the accurate identification of individuals and their parental relationships (Aureli and Schino 2019; Rubenstein et al. 2015; Sundaresan, Fischhoff, Dushoff, and Rubenstein 2007). However, recent advances in non‐invasive genetic sampling and SNP genotyping (Hemstrom et al. 2024; Ramírez‐García et al. 2025; Parker et al. 2025) have made it increasingly feasible to study the mating success of such species using high‐resolution tools for individual identification, parentage assignment, and genetic relatedness inference (Bach et al. 2022; Ekblom et al. 2021; Foroughirad et al. 2019; Kan‐Lingwood et al. 2025, 2026).

Here, we examined inbreeding patterns in the fission–fusion Asiatic wild ass ( *Equus hemionus* ) in the Negev Highlands, Israel, using a non‐invasive genetic approach. As in other fission–fusion equids, high‐ranking males defend territories near water sources, where most breeding occurs (Klingel 1977; Saltz et al. 2000; Sundaresan, Fischhoff, and Rubenstein 2007). Thus, near a water source, there are only a limited number of sites close enough to water where males can establish territories and gain mating success. We asked whether the observed mating‐pair relatedness (MPR) differs from random expectations within annual mating pools, whether inbreeding patterns changed before, during, and after a 2020 artificial water source increase and redistribution that increased the number of territorial males, and whether these changes were associated with mating opportunities involving eight veterans (i.e., males who had reproduced since 2019) versus 33 new reproducing males (i.e., males who had reproduced since 2020).

We also asked whether inbreeding patterns differed spatially between the old and new water sources. We predicted a reduction in inbreeding following the increase and redistribution of water sources because an increasing number of new mating sites near the new water sources would be used by different reproducing males, including males that are genetically less related to females. We predicted increased mating events between less‐related individuals based on extensive evidence of inbreeding costs in wild mammals and female avoidance of mating with familiar males that are likely to be close relatives (Clutton‐Brock and Lukas 2012). Because veteran males can maintain long territorial tenure near water sources, potentially for up to 5 years (Renan et al. 2026), and long‐term territorial use of the old water source was documented in this population (Renan et al. 2015, 2026), we also predicted that the MPR would be higher among mating pairs comprising males associated with this source than among those associated with the new water sources.

## 2 Methods

### 2.1 Study Species and Area

The Asiatic wild ass ( *E. hemionus* ) is a Near Threatened species that inhabits arid and semi‐arid environments across central and eastern Asia, and historically, the Middle East (Kaczensky et al. 2025). In the environments where it occurs, water availability strongly shapes the movement and social organization of Asiatic wild ass individuals (Davidson et al. 2013; Giotto et al. 2015): Females with young foals need to drink daily, whereas non‐lactating females may drink every 3–5 days, leading to changing movement patterns and unstable group composition (Rubenstein 2010). Because males cannot defend or monopolize these unstable female groups, reproducing males defend territories near valuable resources, especially water sources and food patches, where females are likely to occur during the breeding season (April–September) (Bowyer et al. 2020; Boyd et al. 2016; Rubenstein et al. 2015; Saltz et al. 2000).

Females leave their mothers at adolescence and join other non‐stable female groups with no particular grouping pattern or time‐frame limitation; this occurs at approximately 3 years of age in Grevy's zebras but is more variable in wild asses (Moehlman 2002, 2005; Nowzari et al. 2013; Rubenstein 2010; Saltz and Rubenstein 1995). Males also typically remain with their mothers until approximately 3 years of age before joining bachelor groups, although they may depart at younger ages because of expulsion by stallions (Saltz and Rubenstein 1995; Saltz et al. 2000; Tesfai et al. 2021). Because territories are large (estimated at approximately 1–4 km^2^; Giotto et al. 2015; Ziv 2016) and territorial males wander widely to patrol them, bachelor males may occasionally secure matings with unattended females (Kannan et al. 2017). Previous studies showed that territorial Asiatic wild ass males can maintain tenure for up to 7 years and are the main limiting factor affecting variance effective population size (*N*_*ev*_) (Greenbaum et al. 2018; Renan et al. 2015; Saltz et al. 2000). First births occur in May and June, and postpartum oestrous usually occurs within a few weeks after parturition (Saltz et al. 2000). Gestation lasts for 11 months, usually resulting in one foal, or two on rare occasions, and females may give birth in consecutive years, having a total estimated period from oestrous to birth of approximately 12 months (Saltz and Rubenstein 1995).

The Asiatic wild ass was reintroduced to Israel in the early 1980s and 1990s after 11 individuals derived from the Persian wild ass ( *E. hemionus onager* ) and the Turkmenian wild ass ( *E. hemionus kulan* ) were brought to the Hai‐Bar breeding core in the Arava region in 1968 (Saltz and Rubenstein 1995). First reintroductions took place in Makhtesh Ramon, and during the early 1990s, the population naturally expanded to the Negev Highlands (Saltz and Rubenstein 1995). The study area at the Negev Highlands is a protected nature reserve where other wild ungulates such as the Nubian ibex ( *Capra nubiana* ) and Dorcaes gazelles ( *Gazella dorcas* ) occur (Polak et al. 2014; Shkedy and Saltz 2000), but no domestic livestock forage at present. Today, the current minimum Asiatic wild ass population size estimate for the Negev Highlands is approximately 250 individuals, with an increasing trend (Kaczensky et al. 2025).

During the breeding season, the activity of wild ass individuals is concentrated mainly near water sources (Giotto et al. 2015; Saltz et al. 2000). Rainfall in the Negev Highlands is low (average annual precipitation of 95 mm) and occurs mainly from October to March. Strong rainfall events may generate short‐term surface runoff and flow through wadis, and rainwater may also temporarily accumulate in natural cisterns. However, these temporary water sources are unlikely to persist into the summer breeding months because of the long dry season and high evaporation rates (Kidron and Zohar 2010). Therefore, rainfall is not expected to have a direct effect on males' breeding opportunities during the mating season (June–September), and breeding events mainly occur at territories located near stable artificial water sources, which the Israel Nature and Parks Authority (INPA) operates during the summer to support this population. In the Negev Highlands, a single water source was established in the early 1990s (Nezer et al. 2017).

It was recently shown that, although the hybrid ancestry of the Negev Asiatic wild ass inflated the population's initial genetic diversity, the population's heterozygosity and inbreeding coefficient had significantly decreased and increased, respectively, since its reintroduction (Renan et al. 2015; Zecherle et al. 2021). Also, it has been shown that fewer than 25% of all males in this population reproduce (Renan et al. 2015), and that its low *N*_*ev*_ is significantly associated with its strong polygamy, which limits the number of territorial reproducing males (Greenbaum et al. 2018). However, it has also been shown that the population's inbreeding coefficient is low (*F*_IT_ = −0.019), indicating limited inbreeding at the overall population level (Zecherle et al. 2021).

**FIGURE 1:**
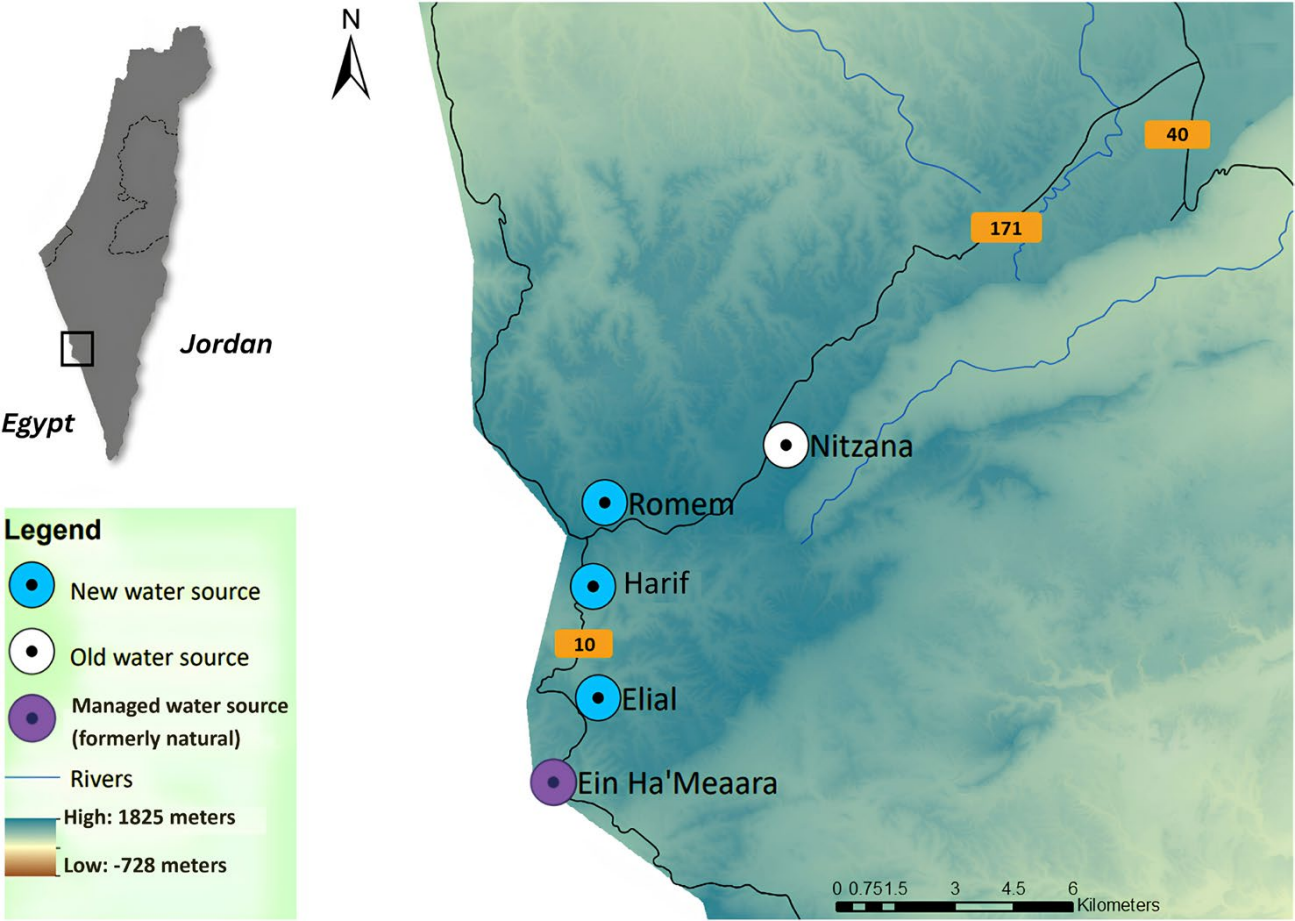
Distribution of the artificial sources providing water to the Asiatic wild ass in the Negev Highlands, Israel, during the breeding seasons. Nitzana: The original water source. Romem, Harif, and Elial: the new water sources. Ein Ha'Meaara: a natural spring that dried out in 2011, following a decrease in ground water levels, and was converted into an artificial water source. Numbers in orange blocks refer to road numbers. Altitude units refer to meters above (high) and below (low) sea level, respectively.

In May 2020, a new intervention plan was implemented in the Negev Highlands to redistribute and increase the number of artificial water sources, where three new artificial water sources were established near the Egyptian border (Figure 1). The original water source previously supporting the population was permanently shut down in September 2020 (Kan‐Lingwood et al. 2026), but the vegetation in the wadi adjacent to it remained abundant, as it was not associated with the water source. Following the increase in the number of watering points, both the number of reproducing males, as measured by the number of adult males that successfully produced foals based on a parentage analysis, and *N*_*ev*_ increased because the number of territorial males near each water point increased (Kan‐Lingwood et al. 2026). These findings underpin our hypothesis regarding changes in inbreeding patterns toward reduced inbreeding following the of artificial water sources supporting breeding sites used by new, different reproducing males.

### 2.2 Sampling, DNA Extraction, Amplification, Sequencing and Genotyping

Sampling was conducted from 2020 to 2024 in the Negev Highlands, Israel, from June to October. Newborn foals appear until September, but we extended the sampling period by 1 month to maximize the chances of capturing late‐born foals and their parents. Given their 11‐month gestation period (Saltz and Rubenstein 1995), foal genotypes, in a given year, reflect matings that occurred in the preceding year. Accordingly, samples collected during 2020–2024 represent matings from 2019 to 2023, corresponding to periods before (2019), during (2020), and after (2021–2023) the 2020 artificial water source increase and redistribution.

Age determination was based on dung size as described in Kan‐Lingwood et al. (2025). The sampling routine was consistent and included sampling the area within 1–4 km from the original and new artificial water sources, as well as collecting samples whenever a female, a bachelors' group, or a solitary male was directly observed between these water sources. The data recorded for each sample during collection is fully described in Kan‐Lingwood et al. (2025).

In addition to samples collected from the wild population of the Negev Highlands, we non‐invasively sampled 10 individuals from the Asiatic wild ass breeding‐core in Hai‐Bar Yotvata (totalling 19 samples, two repeats per individual except one) (Saltz and Rubenstein 1995). These samples included two known half‐sibling foals and their known father and mothers. Genetic sampling of dung was performed for these individuals only when they were directly observed defecating, and two samples were collected from each individual to secure a substitute if one sample was of low quality. These were later used as controls for the genotype filtering process (based on Kan‐Lingwood et al. 2025) and for the parentage analysis (Jones and Wang 2010, further details below). We also included 52 blood or tissue samples from road‐killed individuals within the study area, collected by the INPA rangers during the study period. Sequencing and genotyping were performed by AgriPlex Genomics (Cleveland, OH, USA) using the PlexSeqTM platform, across 625 highly informative SNPs from thousands of SNPs previously identified from captive Asiatic wild asses by Zecherle et al. (2021). They were chosen based on minor allele frequency (MAF) > 0.01 and at least 100 flanking nucleotides, in accordance with AgriPlex Genomics' requirements and the approach described by Kan‐Lingwood et al. (2025).

### 2.3 SNP and Sample Filtering and Genotype Dataset Preparation for Parentage Analysis

From the raw genotype dataset, we filtered low‐quality SNPs and samples and identified ‘unique’ genotypes (i.e., the genetic profile of a single individual) and their recaptures (i.e., repeated samples of a unique genotype) using the analyses described in Kan‐Lingwood et al. (2025). Recaptures of the same individual were defined using the analysis based on the maximum mismatch threshold determined by the recapture simulation analysis (see methods in Kan‐Lingwood et al. 2025). After identifying recaptures, we removed them from the main dataset, retaining only unique genotypes for all downstream analyses.

For the parentage analysis, it was important to properly prepare the genotype datasets of foals (offspring), adult females (candidate mothers), and adult males (candidate fathers) for use as input. Because sampling took place over five consecutive years, some foals were expected to be recaptured as adults in subsequent years. Therefore, a parentage analysis was performed separately for each year, based on the following principles: (1) samples of foals were treated as foals only in the year of sampling; and (2) 3 years later, females were considered candidate mothers based on females' age at sexual maturity (Saltz and Rubenstein 1995), whereas 5 years later, males were considered candidate fathers, based on observation studies showing bachelor males may gain first reproductive success after a 4 years period within bachelor groups (Boyd et al. 2016).

### 2.4 Parentage and Relatedness Analysis

Mating pairs were detected in the genotyping dataset using parentage analysis, in the COLONY 2.0.6.7 program (Jones and Wang 2010). Input parameters were set as described in Kan‐Lingwood et al. (2026). Parental assignments were based on both pairwise and maximum‐likelihood (ML) estimators, and only when the maximum confidence value was received (95% in the pairwise method and 1.00 in the ML method). Assignments based on the ML estimator were preferred if a parental assignment was given with both estimators (Wang and Santure 2009).

We used the genotype dataset of all unique individuals identified in Section 2.3, including foals, adult males, and adult females, as input for a genetic relatedness (*r*) analysis in Coancestry 1.0.1.2 (Wang 2011). This program uses five estimators to calculate pairwise *r*, from which one can be selected based on its strongest correlation with the others (Wang 2011). The resulting *r* values were used in the dyadic analyses described below, including the mating pairs identified from the parentage analysis output. Because marker‐based *r* estimates can be imprecise at the individual level (Taylor 2015), we estimated uncertainty around these values using the standard deviation (SD) of *r* values across all parent–offspring assignments identified by the COLONY parentage analysis, which were considered reliable based on a parentage simulation performed using a dataset genotyped across the same SNPs in Kan‐Lingwood et al. (2026).

### 2.5 Inbreeding Patterns Within Years and Between Years in the Periods Before, During, and After the Artificial Water Source Increase in Number and Redistribution

To understand the general inbreeding levels and tendencies in the wild ass population, we first tested the MPR against a random distribution of *r* among all recorded reproducing females and males in a given year. This analysis was performed for each year separately, as the pool of reproducing males can change year‐to‐year (Saltz et al. 2000). In each year, significantly lower MPR values were interpreted as reduced inbreeding relative to the random *r* pool distribution within the same year, whereas higher values indicated stronger inbreeding. As the MPR distributions deviated from normality, all statistical comparisons were conducted using non‐parametric approaches, including Kolmogorov–Smirnov and permutation tests. Standard deviation (SD) was used as a descriptive measure of overall variation in *r* values, without implying any distributional assumptions.

To test whether observed successful breeding pairs deviated from random expectations in *r*, we constructed year‐specific null models using permutation tests constrained by the observed mating structure. Within each year, female identities and the number of successful breeding events were held constant, while male identities were randomly reassigned among the observed reproducing males. Male assignment preserved the observed reproductive skew by randomly assigning sires from the observed sire pool, with selection probabilities proportional to their observed mating frequencies and with the same sire allowed to be assigned more than once. For each permutation (10,000 iterations), MPR values were extracted from the genetic pairwise *r* matrix, and the median *r* across all simulated pairs was calculated. This generated a null distribution of the expected median *r* under random mating, conditional on the observed demographic constraints. Observed medians (i.e., the MPR medians) were compared to this *r* distribution, and empirical *p*‐values were obtained as the proportion of permuted values equal to or more extreme than the MPR median (in absolute values, to obtain a two‐tailed test).

To also capture differences across the full distribution, as reduced inbreeding may be reflected by a lower frequency of highly genetically related matings even if the median *r* remains similar, we ran a two‐tailed two‐sample Kolmogorov–Smirnov (KS) test. The permutation test and the KS test are complementary, as the permutation test evaluates whether the observed median MPR deviates from the expected median *r* under random mating, conditional on the observed mating structure and reproductive skew, whereas the KS test is sensitive to differences across the entire distribution of MPR values. Therefore, rejection of the null hypothesis by either one or both of these tests may indicate a biologically meaningful difference between observed and random mating patterns.

We used a similar approach to test temporal changes in the MPR following the change in water source distribution by comparing MPR distributions between years. Specifically, we assessed this using one‐tailed permutation tests comparing the median MPR of the baseline year, 2019, with each subsequent year. Mating pairs were pooled across the 2 years, being compared and randomly reassigned to groups of the observed sizes over 10,000 permutations. The differences in *r* medians were then recalculated to generate a null distribution. The *p*‐values were calculated as the proportion of permuted *r* values equal to or more extreme than the observed difference between the *r* medians of the years compared. A one‐tailed, two‐sample KS test was performed here as well, between the 2019 sample and each subsequent year. We used one‐tailed tests for this analysis because we had a clear directional hypothesis, as outlined in the Introduction. Also, for one specific case in which an additional analysis of testing differences in MPR variability was needed to explain the results, we performed permutation tests similarly to the process described for the between‐years median test, only comparing the SD of the MPR between 2023 and the preceding years 2020–2022 as the examined statistic. All analyses were performed using the R v4.4.3 environment (R Core Team 2025).

### 2.6 Differences in MPR Between the Old and New Water Sources

Beyond temporal changes, we also spatially associated inbreeding trends with changes in water source distribution by testing differences in the MPR between the old and new water sources. Mating pairs were assigned to the location of the reproducing male's closest water source within a 3‐km radius during the conception year (Kan‐Lingwood et al. 2026), since males are highly territorial during the breeding season and their locations thus provide a strong indication of where matings occurred (Saltz et al. 2000).

Because water sources strongly influence the Asiatic wild ass's movement and space use (Giotto et al. 2015; Saltz et al. 2000), we also tested whether veteran males had shifted their territories toward the new water sources, suggesting a redistribution of existing reproducing males rather than the establishment of territories by new, additional reproducing males. To address this, we tracked the spatial locations of reproducing males from 2019 to 2024 using faeces recaptures identified via the analyses described in Kan‐Lingwood et al. (2025).

Because not all reproducing males were directly sampled during conception years, the *r* analysis was restricted to comparisons across years rather than annual comparisons. The MPR distributions were compared using a one‐versus‐all approach, in which each water source was compared against the pooled MPR distribution from all other water sources. This resulted in four comparisons: the old water source, Nitzana, versus all other water sources, and each new water source versus all other water sources (including the old): Romem Versus all other water sources, Harif versus all other water sources, and Elial versus all other water sources. These comparisons were tested using two‐tailed permutation and KS tests, as described in Section 2.5.

We also considered whether reproducing males were themselves closely genetically related, which could limit the inbreeding‐reducing benefits of the water source intervention, regardless of the increased mating opportunities (Kan‐Lingwood et al. 2026). This can occur in polygynous systems in which successful mating males produce sons who also attain high reproductive status (Weatherhead and Robertson 1979). To test this, we compared the within‐group *r* between reproducing and non‐reproducing adult males, using a resampling approach. For each male, we calculated his median *r* with all other males within his group. We then calculated the observed median within‐group *r* for reproducing and non‐reproducing males, and the observed difference between them as reproducing minus non‐reproducing males. To assess statistical significance, we generated 1000 bootstrap estimates by randomly selecting one male from each group in each iteration and subtracting the non‐reproducing male's within‐group *r* from the reproducing male's within‐group *r* (Figure S1, Appendix S5). We considered the difference statistically significant when the 95% bootstrap confidence interval did not include zero.

### 2.7 Successful Breeding Events and Genetic Relatedness Between Females and Veteran Versus New Reproducing Males

After testing whether the MPR differed from random expectations within years and examining the changes in inbreeding patterns over time, we next asked whether these patterns could be explained by females' relatedness to veteran versus new reproducing males. Because the water source intervention increased the number of new territorial reproducing males, we specifically asked whether these new males were less genetically related to females than veteran males. We therefore tested: (1) whether females, within each year, were generally more or less genetically related to available new males than to available veteran males; and (2) whether the successful breeding male was more or less related to the female than males in the alternative group. This comparison was used to test whether successful breeding with one male group, rather than the other, was associated with lower *r*.

To test for these patterns, we identified females from the mating‐pair dataset for 2020–2023 for which both veteran and new males were present within the reproducing male pool of a given year (2019 was excluded because it included only veteran males). The test involved a bootstrap approach similar to that described in Section 2.6, with minor modifications based on the level of comparison. For the first question, we compared the female's *r* to available new versus veteran males by repeatedly sampling one male from each group across 1000 iterations and calculating the difference in *r* (new minus veteran). We then bootstrap‐resampled these differences across all cases to estimate the population‐level *r* median and its 95% confidence interval. For the second question, we compared the *r* between each female and the successful breeding male with the *r* between that female and a randomly sampled male from the alternative group across 1000 iterations, generating a percentile‐based 95% confidence interval that was tested as described in Section 2.6.

### 2.8 Ethics Statement

All fieldwork, biological sampling, and research procedures were conducted under permits issued by the INPA, the national authority responsible for wildlife research in Israel (permit numbers 43343 and 43624). All procedures complied with the Israeli Wildlife Protection Law, 1955, and the National Parks, Nature Reserves, National Sites and Memorial Sites Law, 1998.

## 3 Results

### 3.1 Sampling, Sequencing and Genotyping

A total of 2509 faecal samples were collected in the Negev Highlands. Among them, 31 were not extracted due to technical constraints, and 550 failed in the sex‐PCR test (263 showed no band, and 287 had only one or two sex‐PCR runs and did not meet the three sex‐PCR result criteria). Following removal of these low quality samples, a total of 1928 faecal samples were sequenced. With the addition of the 64 tissue samples of road‐killed individuals, 19 samples of the 10 individuals from the Hai‐Bar (as detailed in Section 2.2), and 170 repeated samples of triplicates (see Kan‐Lingwood et al. 2025), the final dataset sent for sequencing included 2181 samples.

Sequencing and genotyping achieved an overall success rate of 87%. Following the genotype filtering procedure (Kan‐Lingwood et al. 2025), 67 SNPs and 126 samples were removed for having less than 66% sequencing success, and 36 SNPs were removed due to an error rate higher than 0.01. Additional 174 repeats of triplicates were removed (see Kan‐Lingwood et al. 2025 for full details). The average error rate across all SNPs after the filtering process was 0.0017 (SD = 0.002).

A total of 1881 genotypes were retained after SNP and sample filtering: 1796 originated from faecal samples collected in the Negev Highlands, 67 from blood and tissue samples, and 18 from Hai‐Bar faecal samples. The recapture‐threshold simulation based on mismatching SNPs, accounting for the SNP error rates, indicated a maximum of 15 mismatching SNPs (Figure S1, Appendix S1). Based on this threshold, 318 unique genotypes were recaptured at least once, ranging from 1 to 16 times per individual, including 6 unique individuals from Hai‐Bar (Figure S2, Appendix S1). After selecting one representative per recaptured individual with the lowest missing data, 821 repeated genotypes were moved to a separate file. Based on the distribution of paired samples by mismatching SNPs (Figure S3, Appendix S1), genotypes with at least 75 mismatching SNPs were considered unique, whereas 502 samples were excluded as they could not be clearly determined as recaptures or unique genotypes (see full method in Kan‐Lingwood et al. 2025). Overall, these results identified 240 unique unrecaptured genotypes, which, together with the 318 recaptured unique genotypes, resulted in 558 unique genotypes across 521 SNPs.

The 558 unique genotypes included 205 adult females, 169 adult males, and 184 foals, distributed at different locations across the study area (Figure S4, Appendix S1). Of these, 46 individuals were sampled both as foals and adults during 2020–2024 (23 females and 23 males, see Table S1, Appendix S2). Based on the unique genotypes and their recaptures during 2020–2024 in the Negev Highlands, we calculated an estimate of the Minimum Number Alive (MNA) (Krebs 1966), which ranged from 156 to 207 per year during 2021–2023, including adults and foals (Table S2, Appendix S2).

### 3.2 Parentage and Relatedness Between Mating Pairs

The parentage analysis conducted on final annual sample sizes for foals, adult males, and females, with consideration of individuals sampled both as foals and adults (Table S1, Appendix S3), resulted in 246 parental assignments, comprising 173 foals that had either a mother or a father or both sampled during the study period. Among these, 73 had both parents sampled. The mating pairs comprised 59 mothers and 41 fathers, totalling 100 reproducing individuals (Figure S1, Appendix S3).

**FIGURE 2:**
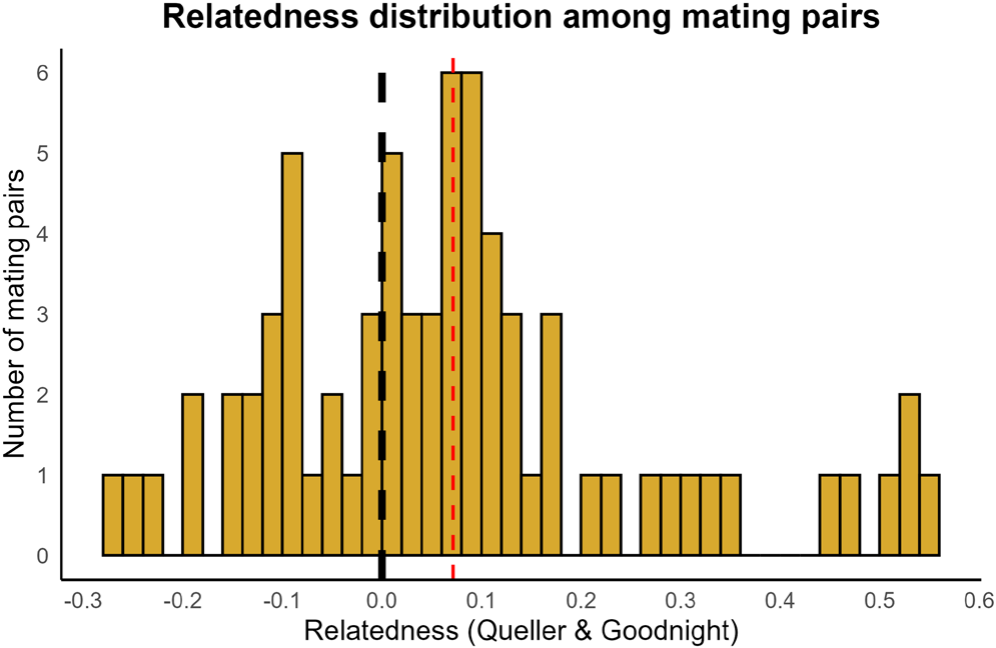
The MPR distribution across all 73 sampled breeding pairs (both parents sampled) was identified from 2019 to 2023. The dashed black line marks where *r* = 0; the dashed red line marks the median MPR = 0.064. Seven mating pairs with *r* around 0.5 may share a parental or full‐sibling relationship.

Among the pairwise *r* estimates, Queller and Goodnight (1989) had the highest correlation with all other estimators (Figure S1, Appendix S4). Using this *r* estimator, the mean and median *r* values between foals and their mothers and fathers across all 246 parental assignments (including foals with only one parent sampled) were 0.498 and 0.497, respectively, with SD = 0.05 (representing the level of uncertainty around the *r* values in our system). The mean and median MPR values among all 73 mating pairs, with both parents sampled across all years, were 0.077 and 0.064, respectively (min = −0.264, max = 0.554, SD = 0.188) (Figure 2; Table S1, Appendix S4).

### 3.3 Inbreeding Patterns Within and Between Years Following the Water Source Increase and Redistribution

**FIGURE 3:**
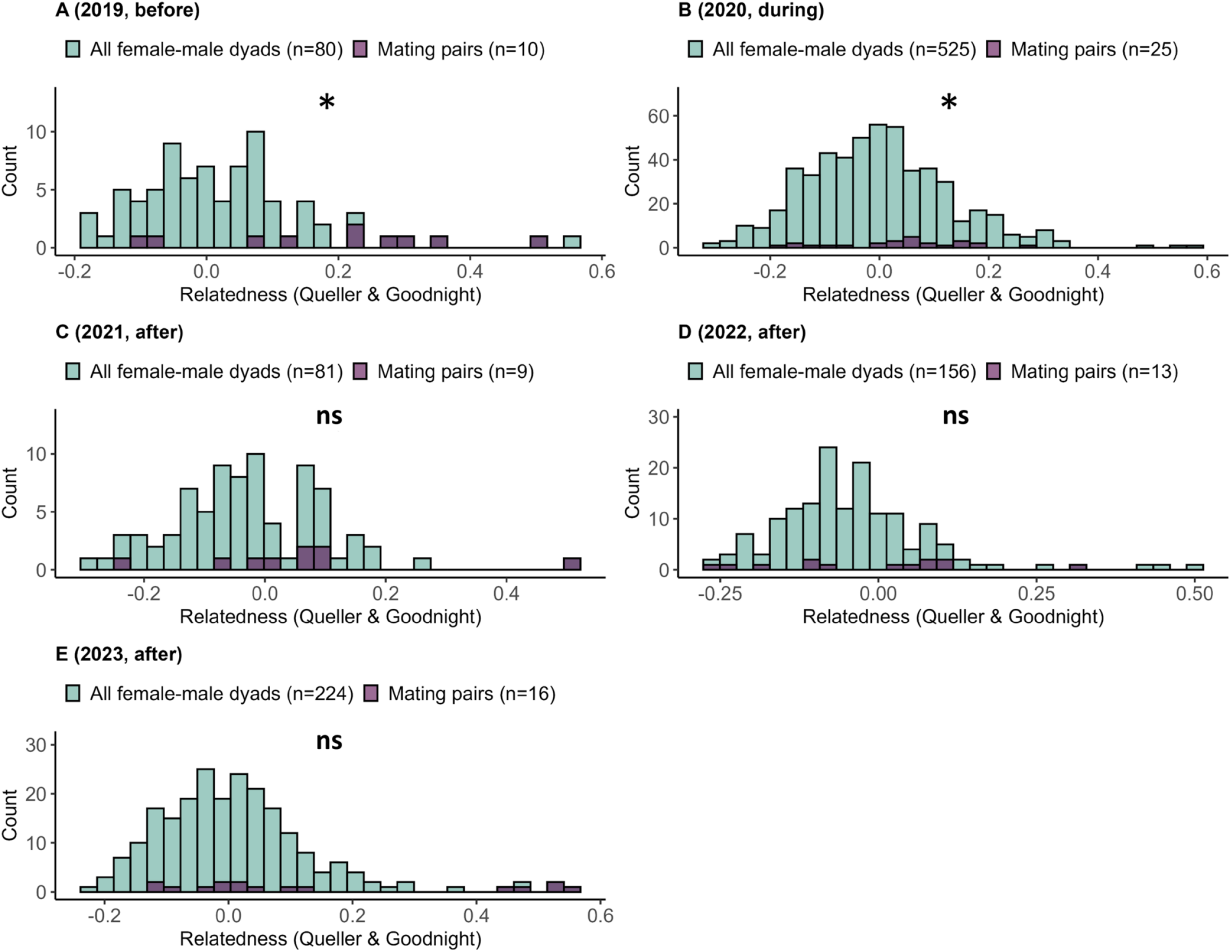
Within‐conception‐year inbreeding patterns results based on two‐sample, two‐tailed KS tests. Pairwise *r* (Queller and Goodnight 1989 estimator) was tested for all available female–male dyads compared to MPR across conception years before, during and after the water source increase and redistribution (A: 2019; B: 2020; C: 2021; D: 2022; E: 2023). Histograms show dyad counts by *r* value; sample sizes per year (*n*) are given in the legend. Significance labels indicate whether the MPR significantly differed from the *r* of all female–male dyads within each year according to the KS test (*p < 0.05, ns: not significant).

Within‐year comparisons between the MPR and random expectations from the annual mating pool showed that the median MPR was significantly higher than expected from random mating in 2019 and 2020 based on two‐tailed permutation tests (2019: observed median = 0.226, random median = 0.007, permutation *p* = 0.0001, SD = 0.19; 2020: observed median = 0.064, random median = −0.003, permutation *p* = 0.0171, SD = 0.12; Table S2, Appendix S4). The MPR distributions were also significantly different from the random distributions in these years based on the two‐tailed KS test (2019: KS *p* = 0.012; 2020: KS *p* = 0.033) (Figure 3A,B). From 2021 to 2023, the median MPR did not differ significantly from random expectations in the permutation tests (2021: observed median = 0.054, random median = −0.032, permutation *p* = 0.3826, SD = 0.20; 2022: observed median = 0.018, random median = −0.060, permutation *p* = 0.9178, SD = 0.17; 2023: observed median = 0.028, random median = −0.009, permutation *p* = 0.3425, SD = 0.26). The KS tests also showed no significant distributional differences (2021: KS *p* = 0.151; 2022: KS *p* = 0.141; 2023: KS *p* = 0.147; Figure 3C–E).

**FIGURE 4:**
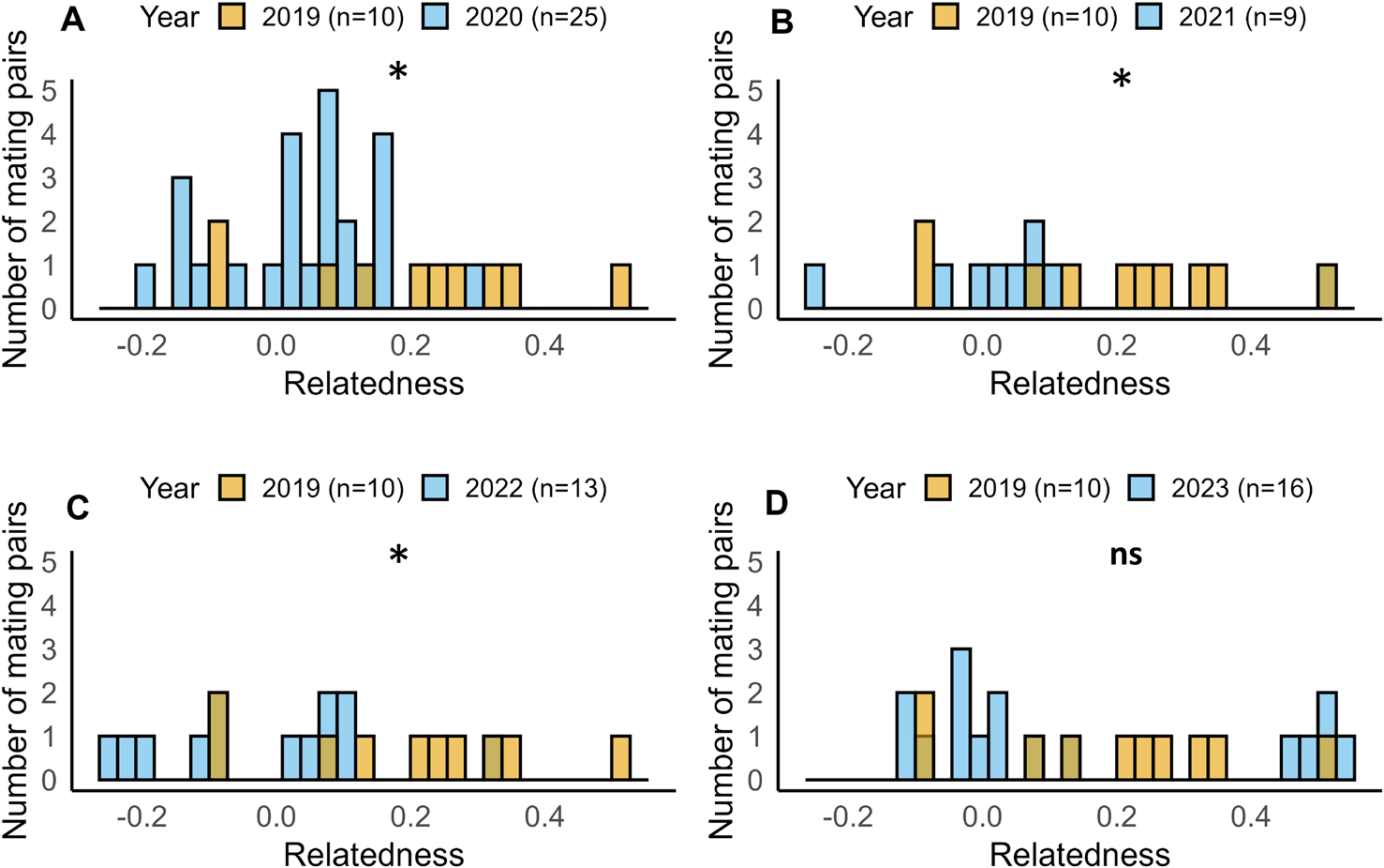
Changes in MPR among successful reproducing pairs before (2019), during (2020), and after (2021–2023) the water source increase and redistribution (years refer to foal conception). Comparisons are shown for (A) 2019 vs. 2020, (B) 2019 vs. 2021, (C) 2019 vs. 2022, and (D) 2019 vs. 2023. Significance based on one‐tailed KS test: *p < 0.05; ns: not significant.

Examining the changes in inbreeding between years, following the water source intervention, showed that the MPR in 2019, before the water source increase, was higher than the MPR in all subsequent years. Permutation tests comparing the MPR in 2019 (median = 0.225) with post‐intervention years showed a significant reduction in the median MPR during the first 3 years following the artificial water source increase and redistribution. Differences in the MPR medians were 0.162, 0.171, and 0.207 for 2020, 2021, and 2022, respectively (one‐tailed permutation *p* = 0.001, 0.035, and 0.019). In 2023, the MPR difference was 0.198, but it was not significant (permutation *p* = 0.111). The comparisons of the full distributions using one‐tailed KS tests detected significant differences between 2019 and 2020 (KS *p* = 0.015; Figure 4A), 2021 (KS *p* = 0.045; Figure 4B), and 2022 (KS *p* = 0.013; Figure 4C), but not 2023 (KS *p* = 0.313; Figure 4D). Although the median MPR remained lower than in 2019 across all post‐intervention years, variability in MPR increased in later years. In 2023, the variability was significantly higher than in 2020 (permutation *p* = 0.004), and although it was also higher than in 2021 and 2022, these differences were not statistically significant (*p* = 0.19 and 0.08, respectively).

### 3.4 Inbreeding Patterns Across Water Sources and Relatedness Among Reproducing Males

**FIGURE 5:**
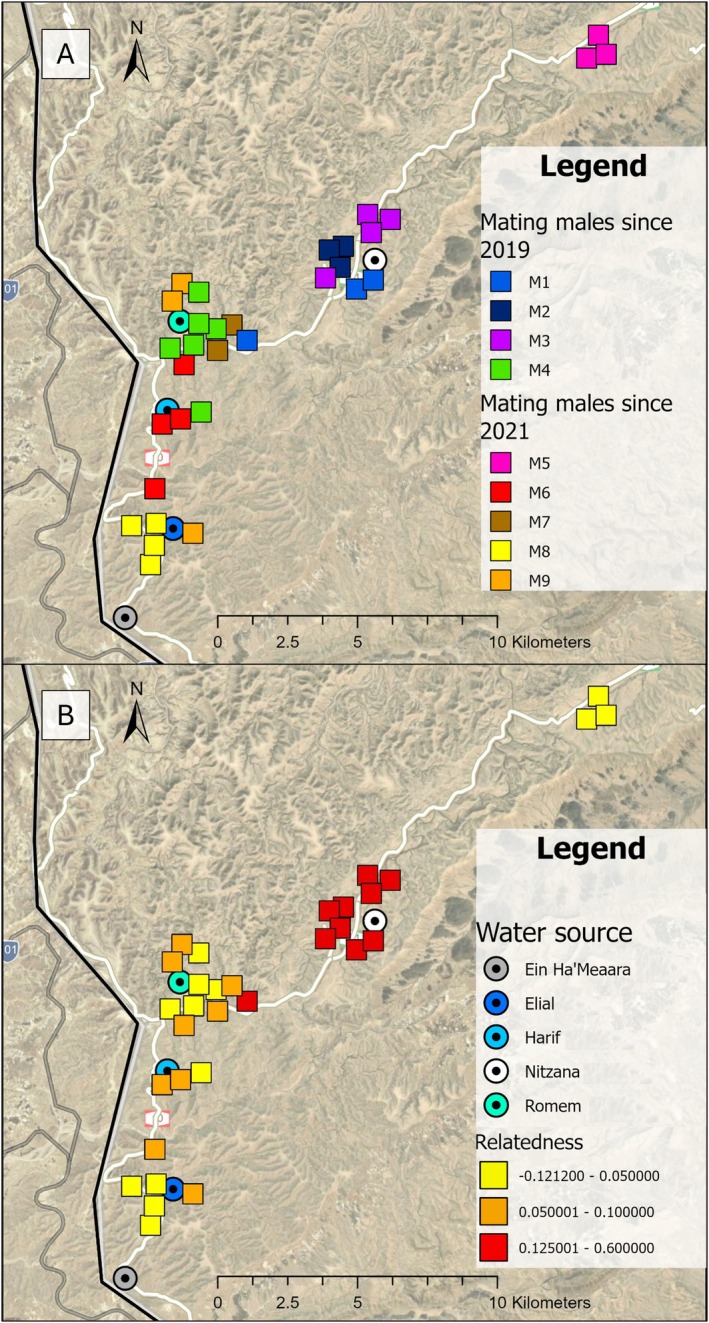
Spatial distribution of nine reproducing Asiatic wild ass males and the MPR with the females they mated with during foal conception years (2019–2023). (A) Recapture locations of the nine reproducing males, with each colour representing a single male (M1–M9). (B) MPR between these males and the females they mated with during foal conception years (2019–2023). Squares indicate the males' locations during conception years, and colours range from higher (red) to lower (yellow) MPR.

A total of 10 reproducing males were sampled during each foal conception year from 2019 to 2023: four reproduced since 2019 (veteran) and six since 2020 (new) (Figure 5). As one reproducing male had only a single sampling during the study period, he was excluded from the spatial analysis, and the MPR values and locations of the other nine reproducing males were examined. The spatial patterns suggested that reproducing males generally maintain similar locations near the same water sources across years: Most veteran males continued to show strong affinity to the old source even after it was allowed to dry up (M1, M2, M3, Figure 5A), whereas males reproducing after 2020 were mainly located near the new sources, despite some inter‐annual fluctuations (e.g., M6–M9, Figure 5A). M4 is a notable exception because although he sired an offspring in 2019, he was sampled only from 2020 onward. Therefore, we can only assume that he held territory at the single old water source in 2019 and later shifted to a new water source after the intervention.

The MPR calculated based on territorial reproducing males' locations during the years of foal conception was significantly higher at the old water source than across all other water sources combined (Nitzana: median *r* = 0.136; all other sources: median *r* = 0.020; Δ median = 0.116; permutation *p* = 0.007; KS *p* = 0.006; Figure 5B). Among successfully mating pairs near the new water source Romem, the MPR was significantly lower than at all other locations combined (Romem: median *r* = −0.018; all other sources: median *r* = 0.086; Δ median = −0.104; permutation *p* = 0.016; KS p = 0.045). No significant differences were found for Harif (median *r* = 0.072; permutation *p* = 0.734; KS *p* = 0.474) or Elial (median *r* = 0.012; permutation *p* = 0.519; KS *p* = 0.742) compared with all other water sources combined.

Testing *r* among reproducing males versus all other non‐reproducing adult males in the population showed no significant difference in within‐group *r*. The observed median within‐group *r* was −0.00575 among reproducing males and 0.00158 among non‐reproducing males. The percentile bootstrap 95% CI overlapped zero (Figure S1, Appendix S5), indicating no significant difference in *r* among reproducing versus non‐reproducing adult males in the population.

### 3.5 Successful Breeding Patterns and Females' Genetic Relatedness to Veteran Versus New Reproducing Males

Across 63 successful breeding events that occurred during 2020–2023, females produced offspring with new males in 87.3% of cases. Overall, available new males were less related to females than available veteran males, with a median *r* difference of −0.051 and a 95% CI that did not overlap zero (−0.0686 to −0.0116, Figure S2, Appendix S5). In 68.3% of cases, females were less likely to be related to the available new males than to the available veteran males. However, comparisons between the male that sired the offspring and the alternative male group did not provide clear evidence that successful breeding events consistently involved the least related available male. Across all cases, successfully breeding males were significantly less related than the alternative male group in 33.3% of cases and significantly more related in 41.3% of cases. Thus, successful breeding events involved new males in most cases, and these new males were overall less related to females than veteran males. However, comparisons with the alternative male group showed that successful breeding events did not consistently involve the least‐related available male group (Figure S2, Appendix S5).

## 4 Discussion

In this study, we examined inbreeding patterns in the fission–fusion, resource‐defence polygynous Asiatic wild ass, and how these inbreeding patterns are associated with changes in access to water sources and, where new breeding males established territories, following water source increase and redistribution (Kan‐Lingwood et al. 2026). By focusing on a fission–fusion species, this study contributes to the current understanding of inbreeding avoidance in equids, which has mainly been examined in harem‐forming species (Berger and Cunningham 1987; Górecka‐Bruzda et al. 2023; Monard and Duncan 1996; Tong et al. 2015). It also expands the current understanding of the effects of habitat configuration on inbreeding patterns in an equid species that is highly dependent on water availability for sociality and reproduction (Kan‐Lingwood et al. 2026; Renan et al. 2026; Saltz et al. 2000) and suggests that changes in resource distribution may contribute to reduced inbreeding without intrusive interventions.

### 4.1 Inbreeding Patterns in the Asiatic Wild Ass Population and Their Changes Following the Artificial Water Source Increase and Redistribution

Within‐year inbreeding patterns, before the water source intervention, showed that the MPR in 2019 was significantly higher than random expectations. We interpret this as the result of veteran males' long‐term tenures around the old water source, with their repeated mating participation over several generations likely making them increasingly genetically related to females reaching sexual maturity (Figure 5B). This pattern is also reflected in the distribution of MPR values. In Figure 3A, for example, the observed MPR distribution included relatively few low‐relatedness mating pairs compared with the distribution of all potential female–male dyads, suggesting that actual mating pairs were shifted away from the lower‐relatedness portion of the available mating pool.

A notable example of a veteran male with an exceptionally long tenure is male 6001, who was GPS‐collared in 2013 and has been recorded as solitary and territorial in previous studies (Giotto et al. 2015; Ziv 2016), as well as in this one. His consistent dominance, as inferred by direct observations and GPS data during 2012–2014 (Giotto et al. 2015; Ziv 2016) and reproductive success (2019–2023) (Table S1, Appendix S4), suggests that territorial Asiatic wild ass males can maintain even 10 years of territorial tenure, longer than the 7 years reported by Saltz et al. (2000). This long‐term tenure, along with other veteran males' participation in mating, may have promoted repeated mating within a limited male mating pool, thereby increasing the MPR before the water source intervention.

In 2020, the MPR remained significantly higher than random mating *r* expectations, indicating that successful breeding events still involved more genetically related matings than expected during the intervention year. The persistence of significantly higher MPR compared to random *r* expectations, within 2020, may reflect a gradual reorganization of the social and spatial structures around the new water sources, which were still in an early transitional phase. In arid ungulates, resource distribution first shapes female movements, and only later allows males to adjust their reproductive strategies to the new landscape (Rubenstein 1989). This tendency is also supported by our data showing that during summer 2020, three new reproducing males were located near the new water sources, while three veteran males continued mating near the old water source with genetically related females (average MPR = 0.124, min = 0.065, max = 0.171).

From 2021 to 2023, the within‐year MPR no longer differed significantly from random expectations. This pattern suggests that the decline in inbreeding after 2020 reflects strong turnover in reproducing males and the increased contribution of new reproducing males to the mating system. Indeed, only four of 38 offspring produced in 2021–2023 were sired by veteran males that is, males who had reproduced since 2019 (one offspring in 2021, two in 2022, and one in 2023; Table S1, Appendix S4). Therefore, the new water sources likely enabled additional males to reproduce by attracting previously non‐reproducing males to establish territories in parallel with veteran reproducing males, thus expanding mating options for females. These findings suggest that inbreeding patterns in this population are context dependent: When breeding was concentrated near a single water source, breeding events were more common among genetically related mating pairs, whereas increased water‐source distribution created additional breeding locations where different males held territories and reproduced, allowing females to disperse and access these new breeding sites, thereby reducing, though not eliminating, inbreeding.

Between‐year comparisons of MPR in 2019 and subsequent years (i.e., temporal trends in inbreeding following the redistribution of water sources) suggest that the mating structure responded dynamically to the redistribution of water sources rather than simply linearly reducing inbreeding. The first 3 years post‐manipulation (2020–2022) showed a clear and consistent decrease in MPR (Figure 4A), associated with the increased mating opportunities of previously excluded males (Kan‐Lingwood et al. 2026). In contrast, despite a reduction in the median MPR in 2023, comparable in magnitude to that observed in earlier post‐increase and redistribution years, the difference from 2019 was not statistically significant. This likely reflects the higher variability observed in 2023, which showed the largest SD and a broader distribution of MPR values, including 5 out of 16 pairs with very high *r* (Figure 4D). Increased variance can reduce statistical power to detect differences in a central tendency, particularly with moderate sample sizes (Cohen 1988). In addition, the KS test has limited sensitivity to differences in variance and tail behaviour, especially under small sample sizes (Baumgartner and Kolassa 2021). Thus, the absence of statistical significance in 2023 does not necessarily indicate a weaker biological effect but may instead reflect the combined influence of increased variability and the reduced ability of the statistical tests to detect such differences. The presence of several highly related mating pairs in 2023 further suggests that, despite overall reductions in inbreeding, mating among close kin still occurs. This may reflect incomplete reduction of inbreeding or context‐dependent constraints on mate choice, potentially involving trade‐offs with other ecological or social factors, although these mechanisms remain to be tested. Together, these results suggest that resource redistribution initially reduced inbreeding by increasing mating opportunities for new adult males, but that over time, this pattern weakened.

One potential cause of this weakening could be increased stability in male territoriality following the intervention, as established males may have gained more consistent reproductive access relative to other male competitors. It could be that some males that reproduced for the first time in 2020 may have subsequently maintained territories across consecutive seasons, thereby increasing the likelihood of repeated mating with related females, who reach sexual maturity at 2 years of age (Saltz and Rubenstein 1995). To evaluate this explanation, we examined temporal changes in the proportions of new reproducing males among all reproducing males during 2020–2023 (excluding 2019, which included only veteran males). Using a two‐proportion *z*‐test with variance‐stabilizing transformation (Freeman and Tukey 1950), as described in Kan‐Lingwood et al. (2026), we found that the proportion of new reproducing males declined significantly from 2020 to 2021 (*Z* = 3.089, *p* = 0.001) and from 2022 to 2023 (*Z* = 1.998, *p* = 0.022) but did not differ between 2021 and 2022 (*Z* = −0.435, *p* = 0.331). These results suggest that the initial expansion of breeding opportunities following water redistribution was followed by reduced turnover among territorial males, potentially contributing to the observed temporal pattern in inbreeding.

### 4.2 Spatial Inbreeding Patterns and Genetic Relatedness Among Reproducing Males

Our spatial results further support that changes in water source abundance and distribution contributed to reduced inbreeding in the population. A significantly higher MPR was found at the original water source (Nitzana) than at the three new water sources (Romem, Harif, and Elial), with Romem having the lowest recorded MPR. Romem was also consistently the water source at which most solitary males were observed, and different reproducing males were detected there across the years (Kan‐Lingwood et al. 2026, and here). This suggests that at this particular water source different reproducing males were present across years, creating greater opportunities for genetically distinct males to establish territories, mate, and contribute to the population's gene pool. Also, our spatial analyses, which examined veteran and new reproducing males' presence near the old and new water sources through the years (Figure 5B), suggest that most veteran males' matings were done near Nitzana and not near the new water sources, supporting our prediction that new territorial reproducing males establish their territories at new water sources and achieve matings with non‐related females.

Moreover, reproducing males were not more genetically related to one another than to other adult males in the population. This finding strongly supports our predictions that the intervention would not only expand males' mating opportunities but also the opportunities to mate with genetically distinct males—a result that will eventually lead to better genetic diversity preservation (Kan‐Lingwood et al. 2026).

### 4.3 Successful Breeding With Less‐Related, New Territorial Reproducing Males

The interpretation that inbreeding patterns depend on ecological context is strengthened by the analysis of successful breeding events with veteran and new reproducing males. When both veteran and new reproducing males were available in a given year, females produced offspring with new males in 87.3% of cases, and these new males were overall less related to them than the veteran males. Thus, although veteran males continued to hold territories near the old water source (Figure 5A), successful breeding events after the water source increase and redistribution were mostly associated with new reproducing males at the new water sources. This is particularly important because females may have been drawn to the original water source, where veteran males hold territories for their familiarity, or to the foraging resources that still existed in veteran territories. This pattern is consistent with reduced inbreeding following spatial reorganization around new water sources (Pusey and Wolf 1996; Pusey 2018), but direct behavioural observations would be needed to determine whether active mate choice, and specifically inbreeding avoidance, contributed to this outcome.

Importantly, however, successful breeding events did not consistently involve the least genetically related male in each case. Among cases in which females produced offspring with new males, the reproducing male was less related than the available veteran group in 33.3% of cases, and more related in 41.3% of cases. This suggests that successful breeding outcomes may also depend on other ecological factors through their effects on dispersal, space‐use patterns, and foraging abilities related to habitat configuration, territory quality, or both (e.g., topography, vegetation cover, proximity to a water source). For example, among Grevy's zebras (Sundaresan et al. 2008) and African wild asses (Tesfai et al. 2021), the reproductive state strongly mediates responses to habitat features, human disturbance, and livestock presence, thereby shaping movement and access to mates, which, in turn, are strongly influenced by environmental conditions.

Other factors may relate to the social conditions that determine dispersal and space‐use patterns. For example, network analyses of Grevy's zebra female social structure showed that females are highly selective in their associations and form stable cliques grouped by reproductive state, such that oestrous females are more likely to associate with particular males and with each other (Rubenstein et al. 2007; Sundaresan, Fischhoff, Dushoff, and Rubenstein 2007). Notably, the multiple mating options that the resource‐defence polygyny system provides for females through breeding sites at males' territories, unlike harem‐forming equid species where a single dominant male largely controls female mating opportunities (Klingel 1975), construct a system in which not only the males' properties (i.e., *r*) but also the territory's qualities are expected to have major impacts on females' breeding sites, and hence, mating decisions and, consequently, on inbreeding (Rubenstein 1993). Therefore, inbreeding and inbreeding avoidance in the Asiatic wild ass are expected to be strongly shaped by both habitat and social factors.

Since the current study estimated inbreeding patterns mainly by using MPR and only slightly by using direct observations, it is difficult to fully understand the Asiatic wild ass's mechanism of inbreeding avoidance. Future work that integrates information on reproducing males' traits, including long‐term reproductive success and their territory's qualities (e.g., topography, vegetation cover, and proximity to water sources), together with females' home ranges and space‐use patterns, will shed light on which factors shape females' space use and successful breeding outcomes, providing a clearer picture of inbreeding avoidance mechanisms in fission–fusion populations, a research field that still seems to lack empirical findings (Boyd et al. 2016).

### 4.4 Conservation and Further Management of the Asiatic Wild Ass Population

As overall inbreeding in the Asiatic wild ass population is relatively low at the population level (Zecherle et al. 2021), the population may not currently be at a clear risk of inbreeding depression. Similar patterns have been reported in other species, where low or even negative inbreeding coefficients occur despite individual‐level inbreeding events (Templeton and Read 1994). Comparative work suggests that this can arise because relatives rarely encounter one another, or because dispersal and mating‐system dynamics limit the actual impact of kin matings at the population level (Batova et al. 2021; Parreira et al. 2020; Pike et al. 2021). In the Asiatic wild ass population, the low inbreeding coefficient could also reflect admixture in the founding population (Zecherle et al. 2021); nevertheless, repeated mating between genetically related individuals could still increase inbreeding over time, especially as the hybridization effect of the founding population is expected to weaken across generations. This is supported by the low inbreeding effective population size (*N*_*ef*_; Zecherle et al. 2021) and the low *N*_*ev*_ of the Negev Desert Asiatic wild ass population that still pose a risk to the population's long‐term persistence (Greenbaum et al. 2018; Renan et al. 2015).

At the same time, the low population‐level inbreeding coefficient may also indicate that other processes, such as space‐use patterns and group or mating structures, shape females' access to different sites where reproduction occurs and, consequently, affect the observed patterns of inbreeding. Therefore, monitoring relatedness at the mating‐pair level is important for detecting emerging inbreeding patterns before they become apparent at the population level and may pose a conservation concern. Periodic changes in the water source distribution, as demonstrated here, may help keep the population's MPR and inbreeding relatively low, with impacts on effective population sizes in the long term (Kan‐Lingwood et al. 2026). However, similar water source changes should be applied cautiously, as other wild or domestic herbivores may also respond to changes in water source availability, with potential consequences for the focal population and ecosystem (Mpalo et al. 2024).

### 4.5 Conservation Implications for Other Species and Systems

Beyond the Asiatic wild ass case study, our findings highlight broader conservation challenges in species, in particular species forming resource‐defence polygyny, where reproduction is spatially constrained by discrete resources that can be monopolized by potential mating males. In such systems, shifts in resource distribution can strongly reshape the effective mating pool by changing where females aggregate and where males can establish territories, thereby influencing MPR and inbreeding. This mechanism may be relevant not only to equids with territorial polygyny, such as the African wild ass ( *E. africanus* ), kiang ( *E. kiang* ), feral donkeys ( *E. asinus* ), and Grevy's zebras ( *E. grevyi* ) (Boyd et al. 2016; Kaczensky et al. 2025; Klingel, 1974; Moehlman 1998; Renan et al. 2018; Rubenstein 1986), but also to other mammals with resource‐defence polygyny mating systems, including European roe deer ( *Capreolus capreolus* ) (Coulon et al. 2006), guanaco ( *Lama guanicoe* ) (Marino et al. 2016), Alpine chamois (*Rupicapra r. rupicapra*) (Cotza et al. 2023), and the endangered Florida bonneted bat ( *Eumops floridanus* ) (Torrez et al. 2020).

By contrast, species forming other mating systems may respond differently to changes in resource availability, and therefore may be less sensitive to resource redistribution in terms of inbreeding. For example, in harem‐forming equids, in which the stallion and its defended females remain in stable social groups when foraging or visiting water sources, resource redistribution may be less likely to directly change the number or identity of potential mating males available to females (Boyd et al. 2016). Thus, the effects of resource availability on inbreeding are expected to depend on how resources shape female space use, male reproductive strategies, and access to breeding locations.

In species forming resource‐defence polygyny, dispersal and resource availability can shape kin encounters and help keep inbreeding low as long as habitats remain connected and landscapes remain permeable (Kaczensky et al. 2008; Renan et al. 2018; Rubenstein et al. 2015; Smith et al. 2022; Sundaresan, Fischhoff, Dushoff, and Rubenstein 2007). However, climate‐driven changes in water and vegetation may constrain movement and space‐use patterns, concentrate individuals around fewer resource points, and impact mating and inbreeding (Della Libera et al. 2023; Kerr 2020). Conversely, increasing the spatial availability of such resources can relax these constraints, expand the pool of breeding males, and reduce the observed MPR. If sustained over time, such increases in the MPR may reduce effective population sizes and genetic diversity, thereby increasing extinction risk in small or isolated populations. Our results, therefore, suggest that management actions that deliberately target resource distribution, and spatially explicit management of key resources, such as water, can serve as a practical tool for mitigating genetic erosion and helping prevent future loss of genetic diversity in fragmented arid landscapes.

## Author Contributions

N.Y.K.‐L., S.B.‐D., A.B., A.R.T., and D.I.R. developed the idea of this research. S.B.‐D. designed the genetic monitoring approach. N.Y.K.‐L. conducted the field work (genetic and direct observations), collected the samples, and processed them with N.S. N.Y.K.‐L., A.B., A.R.T., and S.B.‐D. designed the analyses. N.Y.K.‐L. and A.B. wrote and executed the methods for the study's analyses. N.Y.K.‐L. wrote the original manuscript and revised it with S.B.‐D. All authors commented on the manuscript.

## Funding

This research was supported by the United States–Israel Binational Science Foundation (BSF) grant no. 2011384 (resubmission no. 2017288) awarded to Prof. Shirli Bar‐David, Prof. Daniel I. Rubenstein, Prof. Alan R. Templeton, and Prof. Amos Bouskila, and the Mohamed bin Zayed Species Conservation Fund (grant 232532597) awarded to Mrs. Noa Yaffa Kan‐Lingwood, Mrs. Naama Shahar, and Prof. Shirli Bar‐David.

## Conflicts of Interest

The authors declare no conflicts of interest.

## Supporting information


**Appendix S1:** SNP mismatch simulation, recapture distribution, and spatial locations of unique genotypes (*n* = 558).
**Figure S1:** Simulation result for the maximum mismatching SNPs between recaptures. The simulation was based on the calculated error rate per SNP, resulting with a maximum of 15 mismatching SNPs (1,000,000 runs) (Kan‐Lingwood et al. 2025).
**Figure S2:** Distribution of individuals (*n* = 570 unique genotypes) by the number of times each individual was captured.
**Figure S3:** Distribution of sample dyads by mismatching SNPs. This distribution was used to detect unique genotypes and recaptures. Based on the distribution, the ‘ambiguous area’ was defined from 15 to 75 mismatching SNPs between sample pairs, shown in the small square.
**Figure S4:** Spatial distribution of 558 genotypes sampled from 2020 to 2024 in the Negev Highlands. The study area focuses where three new water sources were established (Elial, Harif and Romem) and the single, original water source which supported the Asiatic wild ass population (Nitzana) was dried out in 2020. Ein Ha'Meaara is a natural spring that dried out in 2011, following a decrease in ground water levels, and was converted into an artificial water source.
**Appendix S2:** Individuals captured both as foals and adults in 2020–2024 and Minimum Number Alive (MNA) estimation.
**Table S1:** Details of individuals (genotypes) captured both as foals and adults from 2020 to 2024.
**Table S2:** Minimum Number Alive (MNA) estimation. MNA was calculated from sampling records of genetically identified individuals by counting each individual as alive in each year between its first and last detection (Krebs 1966). Annual counts were summarized by sex/age class, with juveniles defined as individuals younger than 1 year (foals). Because MNA is based on detection histories, estimates for the first and last years are less reliable than central years. In general, MNA should be interpreted as conservative minimum counts rather than full population‐size estimates.
**Appendix S3:** Parentage analysis inputs and additional results.
**Table S1:** Annual sample sizes of genotype data used as inputs for the parentage analysis (2019–2023). The number of offspring was determined by the number of individuals less than 1 year old sampled in the given year. The numbers of mother and father candidates were based on adult females and males sampled each year, plus individuals that were sampled as juveniles during the study period and reached sexual maturity in the given year (3 years for females and five for males; Saltz and Rubenstein 1995). Because no male sampled as a foal reached sexual maturity during the study period, the number of candidate fathers remained constant across the years.
**Figure S1:** Annual parentage results by category. The graph describes the sample sizes of offspring (orange), mothers (purple), fathers (red), and total mating pairs (turquoise). The data includes offspring for which both parents were sampled and offspring for which only one parent was sampled (mother or father).
**Appendix S4:** Relatedness coefficient correlations and mating‐pair relatedness (MPR) results.
**Figure S1:** Sum of correlations between different pairwise genetic relatedness estimators. The estimators were tested using the Coancestry program (Wang 2011), and included: Li et al. (1993), Lynch & Ritland (1999), Queller and Goodnight (1989), Ritland (1996), and Wang (2002). The Queller and Goodnight (1989) estimator—which assesses relatedness based on allele frequency deviations from the population mean—showed the highest correlation with all other estimators. Li et al.'s (1993) method relies on a similarity index, while Ritland's (1996) estimator uses a multi‐locus approach weighted by the inverse of sampling variances. The Lynch & Ritland (1999) and Wang (2002) estimators are evaluated using simulated, non‐pedigreed populations, assuming no inbreeding and including only simple relative classes.
**Table S1:** Parentage and mating‐pair relatedness (MPR) results for all 73 Asiatic wild ass pairs reproducing in 2019–2023.
**Table S2:** Within‐year permutation results of MPR versus random mating pairs from 2019 to 2023.
**Appendix S5:** Relatedness contrasts associated with males' reproductive status and females' mating opportunities.
**Figure S1:** Bootstrap distribution of relatedness (*r*) differences between reproducing and non‐reproducing males. Histogram of 1000 bootstrap estimates of the difference in median within‐group *r* (Queller & Goodnight estimator), presented as reproducing minus non‐reproducing. Each bootstrap value was obtained by randomly selecting one male from each group, calculating his median relatedness to all other males within his group, and subtracting the non‐reproducing value from the reproducing value. We considered the difference statistically significant when the 95% bootstrap confidence interval did not include zero. Median within‐group *r* was *r* = −0.006 among reproducing males (*n* = 41) and r = 0.002 among non‐reproducing males (*n* = 127).
**Figure S2:** Distribution of relatedness (r) differences to new versus veteran males from 2020 to 2023. Each bar represents the number of females for which a difference in median relatedness between females and available new and veteran males was counted in a given year, calculated as new minus veteran. Negative values indicate a lower r to new males than to veteran males. The dashed vertical red line marks zero as the baseline, the dashed vertical blue line marks the overall observed median difference across all cases, and the dotted grey lines mark the 95% bootstrap CI. Across 63 female‐year cases, the overall observed median difference was −0.051, with a 95% bootstrap CI that did not overlap zero (−0.0686 to −0.0116), indicating that females were overall less genetically related to new than to veteran reproducing males.

## Data Availability

The genotype dataset and original code that support the findings of this study are openly available in the ‘Figshare’ repository at https://figshare.com/s/1ba11a21b002860aeed8 (private link).
