# Supplementary material for: Inbreeding in a Fission–Fusion Equid Decreases Following an Increase in the Number and Redistribution of Water Sources
Source: Mol Ecol. 2026 Aug 17;35(16):e70516. doi: 10.1111/mec.70516 (PMC13482000; doi:10.1111/mec.70516)
Supplement: Supplementary file 1 [file MEC-35-e70516-s001.docx]

**Supplemental Information for:**

**Inbreeding in a fission–fusion equid decreases following increase in the number and redistribution of water sources**

Noa Yaffa Kan-Lingwood, Alan R. Templeton, Naama Shahar, Daniel I. Rubenstein, Amos Bouskila, Shirli Bar-David

**Table of Contents:**

| **Appendix S1. SNP mismatch simulation, recapture distribution, and spatial locations of unique genotypes (n = 558)** | Page 1 |
| --- | --- |
| **Appendix S2. Individuals captured both as foals and adults in 2020–2024 and Minimum Number Alive (MNA) estimation** | Page 4 |
| **Appendix S3. Parentage analysis inputs and additional results** | Page 7 |
| **Appendix S4. Relatedness coefficient correlations and mating-pair relatedness (MPR) results** | Page 9 |
| **Appendix S5. Relatedness contrasts associated with males' reproductive status and females' mating opportunities** | Page 14 |

# **Appendix S1. SNP mismatch simulation, recapture distribution, and spatial locations of unique genotypes (n = 558)**

**Figure S1. Simulation result for the maximum mismatching SNPs between recaptures.** The simulation was based on the calculated error rate per SNP, resulting with a maximum of 15 mismatching SNPs (1,000,000 runs) (Kan-Lingwood et al., 2025).

**Figure S2.** **Distribution of** **individuals (n=570 unique genotypes) by the number of times each individual was captured.**

**Figure S3.** **Distribution of sample dyads by mismatching SNPs.** This distribution was used to detect unique genotypes and recaptures. Based on the distribution, the "ambiguous area" was defined from 15–75 mismatching SNPs between sample pairs, shown in the small square.

**Figure S4.** **Spatial distribution of 558 genotypes sampled from 2020–2024 in the Negev Highlands.** The study area focuses where three new water sources were established (Elial, Harif and Romem) and the single, original water source which supported the Asiatic wild ass population (Nitzana) was dried out in 2020. Ein Ha'Meaara is a natural spring that dried out in 2011, following a decrease in ground water levels, and was converted into an artificial water source.

# **Appendix S2. Individuals captured both as foals and adults in 2020–2024 and Minimum Number Alive (MNA) estimation**

**Table S1.** **Details of individuals (genotypes) captured both as foals and adults from 2020–2024.**

| **Main ID** | **Recapture samples** | **Sex** | **Foal year** | **Adult year(s)** |
| --- | --- | --- | --- | --- |
| 1536 | 1536;3081;3082 | M | 2020 | 2023 |
| 1614 | 1614;1743;1899;2136;2494 | F | 2020, 2022 | 2021 |
| 1615 | 1615;1784;2179;2180;3576 | F | 2020 | 2021, 2024 |
| 1661 | 1661;1902;2679;1670 | F | 2020 | 2021, 2022 |
| 1666 | 1666;1740;2184;3750;3754 | F | 2020 | 2021, 2024 |
| 1713 | 1713;2718 | M | 2020 | 2022 |
| 1715 | 1715;1923;3563 | F | 2020 | 2021, 2024 |
| 1831 | 1754;1831;2549 | M | 2020, 2021 | 2022 |
| 3436 | 1780;2496;3436;3872 | F | 2020 | 2022, 2023, 2024 |
| 1906 | 1906;2392;2423;2804 | M | 2021 | 2022 |
| 3257 | 1952;1969;2050;2981;3257;3535  3541;3652;3663;3675;3847 | M | 2021 | 2023, 2024 |
| 1968 | 1968;2405;2407 | M | 2021 | 2022 |
| 1980 | 1980;3988 | F | 2021 | 2024 |
| 3453 | 2081;2238;2408;3453;2211 | F | 2021 | 2022, 2023 |
| 2100 | 2100;2729;2738;2896 | M | 2021 | 2022, 2023 |
| 3508 | 2139;2892;3508 | M | 2021 | 2023 |
| 2285 | 2285;3282;3742 | F | 2021 | 2023, 2024 |
| 2300 | 2300;3055;3056;3084 | F | 2021 | 2023 |
| 2302 | 2302;2532;3559 | M | 2021 | 2022, 2024 |
| 2349 | 2332;2501;3970;2349;2327 | F | 2021, 2022 | 2024 |
| 2422 | 2421;3891;2422 | M | 2022 | 2024 |
| 3246 | 2426;3223;3246 | M | 2022 | 2023 |
| 2688 | 2485;2686;3699;3700;3705;2688 | M | 2022 | 2024 |
| 3500 | 2490;2509;2693;2886;3425  3430;3500;3515 | M | 2022 | 2023 |
| 3154 | 2518;2685;2739;3154 | M | 2022 | 2023 |
| 2558 | 2558;3324 | M | 2022 | 2023 |
| 3083 | 2560;2616;3052;3054;3083;3086 | M | 2022 | 2023 |
| 3370 | 2592;3370;3683;3771;8009 | F | 2022 | 2023, 2024 |
| 8077 | 2595;2621;8077 | F | 2022 | 2024 |
| 3208 | 2629;3208 | F | 2022 | 2023 |
| 8051 | 2703;8051 | M | 2022 | 2024 |
| 2309 | 2708;3960;2309 | M | 2021 | 2022, 2024 |
| 1782 | 2749;1782 | F | 2020 | 2022 |
| 2758 | 2758;2767;3122;3131 | F | 2022 | 2023 |
| 2993 | 2993;3214;3443;3451;3555 | M | 2023 | 2024 |
| 3529 | 2996;3529 | F | 2023 | 2024 |
| 3048 | 3048;3087;3546 | F | 2023 | 2024 |
| 3155 | 3150;3155;3849 | F | 2023 | 2024 |
| 3179 | 3179;3618;3621 | F | 2023 | 2024 |
| 3219 | 3219;3617 | M | 2023 | 2024 |
| 3359 | 3359;3975 | M | 2023 | 2024 |
| 3395 | 3395;3681 | F | 2023 | 2024 |
| 3403 | 3403;3600;3856 | F | 2023 | 2024 |
| 3423 | 3423;3429;3999 | F | 2023 | 2024 |
| 3438 | 3438;3869 | M | 2023 | 2024 |
| 3444 | 3444;3445;3914;3925;3928 | M | 2023 | 2024 |

**Table S2. Minimum Number Alive (MNA) estimation**. MNA was calculated from sampling records of genetically identified individuals by counting each individual as alive in each year between its first and last detection (Krebs, 1966). Annual counts were summarized by sex/age class, with juveniles defined as individuals younger than one year (foals). Because MNA is based on detection histories, estimates for the first and last years are less reliable than central years. In general, MNA should be interpreted as conservative minimum counts rather than full population-size estimates.

| **Year sampled** | **Adult males** | **Adult females** | **Juveniles (foals)** | **Unidentified** | **Total alive** |
| --- | --- | --- | --- | --- | --- |
| 2020 | 18 | 24 | 18 | 2 | 62 |
| 2021 | 51 | 76 | 51 | 0 | 178 |
| 2022 | 61 | 61 | 34 | 0 | 156 |
| 2023 | 72 | 95 | 40 | 0 | 207 |
| 2024 | 75 | 81 | 52 | 0 | 208 |

# **Appendix S3. Parentage analysis inputs and additional results**

**Table S1. Annual sample sizes of genotype data used as inputs for the parentage analysis (2019–2023).** The number of offspring was determined by the number of individuals less than one year old sampled in the given year. The numbers of mother and father candidates were based on adult females and males sampled each year, plus individuals that were sampled as juveniles during the study period and reached sexual maturity in the given year (three years for females and five for males; Saltz & Rubenstein, 1995). Because no male sampled as a foal reached sexual maturity during the study period, the number of candidate fathers remained constant across the years.

| **Conception year** | **Offspring** | **Mother candidates** | **Father candidates** | **Total** |
| --- | --- | --- | --- | --- |
| 2019 | 20 | 192 | 161 | 373 |
| 2020 | 50 | 192 | 161 | 401 |
| 2021 | 30 | 192 | 161 | 380 |
| 2022 | 49 | 200 | 161 | 406 |
| 2023 | 47 | 207 | 161 | 410 |

**Figure S1.** **Annual parentage results by category**. The graph describes the sample sizes of offspring (orange), mothers (purple), fathers (red), and total mating pairs (turquoise). The data includes offspring for which both parents were sampled and offspring for which only one parent was sampled (mother or father).

# **Appendix S4. Relatedness coefficient correlations and mating-pair relatedness (MPR) results**

**Figure S1. Sum of correlations between different pairwise genetic relatedness estimators.** The estimators were tested using the Coancestry program (Wang, 2011), and included: Li et al. (1993), Lynch & Ritland (1999), Queller & Goodnight (1989), Ritland (1996), and Wang (2002). The Queller & Goodnight (1989) estimator—which assesses relatedness based on allele frequency deviations from the population mean—showed the highest correlation with all other estimators. Li et al.’s (1993) method relies on a similarity index, while Ritland’s (1996) estimator uses a multi-locus approach weighted by the inverse of sampling variances. The Lynch & Ritland (1999) and Wang (2002) estimators are evaluated using simulated, non-pedigreed populations, assuming no inbreeding and including only simple relative classes.

**Table S1.** **Parentage and mating-pair relatedness (MPR) results for all 73 Asiatic wild ass pairs reproducing in 2019–2023.**

|  | Foal ID | Mother ID | Father ID | Conception year | Relatedness among mating pairs (MPR) |
| --- | --- | --- | --- | --- | --- |
| 1 | 1536 | 3238 | 1515 | 2019 | -0.086 |
| 2 | 1542 | 3046 | 1515 | 2019 | -0.0909 |
| 3 | 1604 | 2171 | 6001 | 2019 | 0.5088 |
| 4 | 1614 | 3799 | 1834 | 2019 | 0.2773 |
| 5 | 1661 | 3845 | 1660 | 2019 | 0.216 |
| 6 | 1666 | 3167 | 3165 | 2019 | 0.0694 |
| 7 | 1713 | 2497 | 1684 | 2019 | 0.1273 |
| 8 | 1782 | 2747 | 3851 | 2019 | 0.3103 |
| 9 | 1831 | 3029 | 3165 | 2019 | 0.2353 |
| 10 | 3085 | 3499 | 1895 | 2019 | 0.3481 |
| 11 | 1838 | 2687 | 3649 | 2020 | -0.0648 |
| 12 | 1840 | 1848 | 2096 | 2020 | 0.1703 |
| 13 | 1870 | 1842 | 2074 | 2020 | 0.0716 |
| 14 | 1879 | 1863 | 2754 | 2020 | 0.1152 |
| 15 | 1901 | 1903 | 1684 | 2020 | 0.0464 |
| 16 | 1906 | 2032 | 3229 | 2020 | 0.0201 |
| 17 | 1934 | 1914 | 2098 | 2020 | -0.1306 |
| 18 | 1935 | 4072 | 3230 | 2020 | 0.1712 |
| 19 | 1965 | 3035 | 8014 | 2020 | 0.0105 |
| 20 | 1974 | 3194 | 3165 | 2020 | 0.0933 |
| 21 | 1980 | 1778 | 3992 | 2020 | -0.1953 |
| 22 | 1995 | 2643 | 2732 | 2020 | -0.011 |
| 23 | 2088 | 3195 | 3091 | 2020 | 0.2855 |
| 24 | 2150 | 3442 | 8047 | 2020 | 0.0216 |
| 25 | 2250 | 2239 | 1834 | 2020 | 0.0819 |
| 26 | 2288 | 2282 | 1772 | 2020 | 0.1357 |
| 27 | 2300 | 2089 | 3819 | 2020 | -0.1044 |
| 28 | 2309 | 1701 | 2269 | 2020 | -0.1536 |
| 29 | 2347 | 2354 | 2098 | 2020 | 0.0151 |
| 30 | 2349 | 2324 | 1590 | 2020 | 0.0654 |
| 31 | 2353 | 2326 | 2875 | 2020 | 0.0731 |
| 32 | 2361 | 2998 | 1834 | 2020 | 0.1608 |
| 33 | 3257 | 3247 | 2098 | 2020 | 0.1588 |
| 34 | 3496 | 2176 | 3229 | 2020 | 0.0638 |
| 35 | 3508 | 2125 | 3080 | 2020 | -0.1426 |
| 36 | 2378 | 3401 | 2632 | 2021 | 0.1035 |
| 37 | 2545 | 1903 | 3886 | 2021 | -0.0079 |
| 38 | 2613 | 2606 | 1891 | 2021 | 0.0544 |
| 39 | 2681 | 2092 | 8014 | 2021 | 0.0716 |
| 40 | 2758 | 2171 | 6001 | 2021 | 0.5088 |
| 41 | 2772 | 3248 | 2117 | 2021 | 0.0844 |
| 42 | 2774 | 2466 | 2875 | 2021 | -0.0579 |
| 43 | 3500 | 2126 | 2307 | 2021 | 0.0117 |
| 44 | 8077 | 1825 | 8074 | 2021 | -0.2441 |
| 45 | 2993 | 2970 | 2319 | 2022 | -0.0827 |
| 46 | 3020 | 3035 | 6001 | 2022 | 0.0451 |
| 47 | 3045 | 1914 | 1515 | 2022 | 0.1089 |
| 48 | 3047 | 3046 | 1588 | 2022 | -0.2354 |
| 49 | 3048 | 1943 | 3038 | 2022 | -0.096 |
| 50 | 3219 | 3741 | 3230 | 2022 | -0.2637 |
| 51 | 3243 | 3238 | 3014 | 2022 | 0.0182 |
| 52 | 3288 | 3107 | 1884 | 2022 | 0.0836 |
| 53 | 3353 | 2687 | 1891 | 2022 | -0.1124 |
| 54 | 3375 | 3247 | 3014 | 2022 | 0.108 |
| 55 | 3423 | 3477 | 3992 | 2022 | 0.3268 |
| 56 | 3461 | 2998 | 2461 | 2022 | 0.0892 |
| 57 | 3495 | 3498 | 2269 | 2022 | -0.1965 |
| 58 | 3597 | 3477 | 3888 | 2023 | -0.0813 |
| 59 | 3665 | 2088 | 3091 | 2023 | 0.5257 |
| 60 | 3670 | 3238 | 2632 | 2023 | 0.447 |
| 61 | 3751 | 2239 | 3229 | 2023 | -0.1212 |
| 62 | 3757 | 3756 | 2074 | 2023 | -0.1127 |
| 63 | 3762 | 1538 | 6001 | 2023 | 0.5234 |
| 64 | 3766 | 3167 | 8047 | 2023 | -0.0429 |
| 65 | 3835 | 2195 | 8047 | 2023 | -0.0183 |
| 66 | 3862 | 2106 | 2095 | 2023 | 0.5544 |
| 67 | 3876 | 2032 | 3229 | 2023 | 0.0201 |
| 68 | 3900 | 1785 | 2307 | 2023 | 0.4771 |
| 69 | 3984 | 3255 | 1771 | 2023 | 0.0904 |
| 70 | 8053 | 3633 | 1588 | 2023 | 0.1286 |
| 71 | 8054 | 8062 | 1884 | 2023 | 0.036 |
| 72 | 8060 | 3561 | 3819 | 2023 | -0.0224 |
| 73 | 8078 | 8071 | 3038 | 2023 | 0.0059 |

**Table S2.** **Within-year permutation results of MPR versus random mating pairs from 2019–2023.**

| **Year** | **Observed** | **Random median** | **permutation**  **p-value** | **N mating pairs** | **Standard deviation** |
| --- | --- | --- | --- | --- | --- |
| 2019 | 0.226 | 0.007 | 0.0001 | 10 | 0.19 |
| 2020 | 0.064 | -0.003 | 0.0171 | 25 | 0.12 |
| 2021 | 0.054 | -0.032 | 0.3826 | 9 | 0.2 |
| 2022 | 0.018 | -0.06 | 0.9178 | 13 | 0.17 |
| 2023 | 0.028 | -0.009 | 0.3425 | 16 | 0.26 |

# **Appendix S5. Relatedness contrasts associated with males' reproductive status and females' mating opportunities**

**Figure S1. Bootstrap distribution of relatedness (*r*) differences between reproducing and non-reproducing males.** Histogram of 1,000 bootstrap estimates of the difference in median within-group *r* (Queller & Goodnight estimator), presented as reproducing minus non-reproducing. Each bootstrap value was obtained by randomly selecting one male from each group, calculating his median relatedness to all other males within his group, and subtracting the non-reproducing value from the reproducing value. We considered the difference statistically significant when the 95% bootstrap confidence interval did not include zero. Median within-group *r* was *r* = −0.006 among reproducing males (n = 41) and *r* = 0.002 among non-reproducing males (n = 127).

**Figure S2. Distribution of relatedness (*r*) differences to new versus veteran males from 2020–2023.** Each bar represents the number of females for which a difference in median relatedness between females and available new and veteran males was counted in a given year, calculated as new minus veteran. Negative values indicate a lower *r* to new males than to veteran males. The dashed vertical red line marks zero as the baseline, the dashed vertical blue line marks the overall observed median difference across all cases, and the dotted gray lines mark the 95% bootstrap CI. Across 63 female-year cases, the overall observed median difference was -0.051, with a 95% bootstrap CI that did not overlap zero (-0.0686 to -0.0116), indicating that females were overall less genetically related to new than to veteran reproducing males.
